# Association between microRNA 25 expression in serum and lung cancer: A protocol for systematic review and meta-analysis

**DOI:** 10.1097/MD.0000000000020263

**Published:** 2020-05-15

**Authors:** Peng Luo, Feng Qiao, Peng-Hui Dou, Shu-Min Li, Tian-Lu Zhang, Yu-Tong Xing, Gang Zhou, Su-Kun Xu, Jia-Bin Sun

**Affiliations:** a Department of Cardiothoracic Surgery; b Department of Chemotherapy and Radiotherapy Department; c Department of Respiratory Medicine, First Affiliated Hospital of Jiamusi University, Jiamusi; d Department of Cardiothoracic Surgery, Xiamen Fifth Hospital, Xiamen; e Department of Cardiothoracic Surgery, Jiamusi Hongda Hospital; f Department of Chest Surgery, Jiamusi Tumor Hospital; g Department of Critical Medicine, First Affiliated Hospital of Jiamusi University, Jiamusi, China.

**Keywords:** association, case-controlled study, lung cancer, microRNA 25

## Abstract

**Background:**

This study aims to identify the association between microRNA 25 (mRNA 25) expression in serum and lung cancer (LC).

**Methods:**

This planned study will cover all eligible case-controlled studies that report association between mRNA 25 expression in serum and LC. It will include published studies from inception to the present in Cochrane Library, PUBMED, EMBASE, Web of Science, Allied and Complementary Medicine Database, VIP database, and China National Knowledge Infrastructure regardless language and geographical location. We will also search other sources, such as conference abstracts and reference lists of related known studies and experts in the domain consulted to avoid missing potential studies. Two contributors will independently examine and select studies, collect all necessary data, and judge study quality for all included studies. We will perform statistical analysis using RevMan V.5.3 software and Stata V.12.0 software.

**Results:**

This study will summarize current evidence to present first systematic review of research on the association between mRNA 25 expression in serum and LC.

**Conclusion:**

This study will present comprehensive evidence to determine whether mRNA 25 expression in serum is associated with LC, and will provide helpful evidence for the future studies.

**Systematic review registration:**

INPLASY202040056.

## 1 Introduction

Lung cancer (LC) is one of the most commonly diagnosed cancers (about 11.6% of all cancer cases) around the world.^[1,2,3]^ It is also the leading cause of cancer (about 18.4% of all cancer deaths) mortality globally.^[4,5]^ It was reported about 733,000 new cases and 610,000 deaths in China in 2015.^[6,7]^ Patients with such disorder often manifest as cough (often with blood), chest pain, wheezing, and weight loss.^[8,9,10,11]^ Several risk factors may cause this disease, such as smoking, exposure to secondhand smoke, radon gas, asbestos and other carcinogens, and family history of LC.^[12,13,14,15,16]^ Although a variety of treatments have reported to treat LC, its 5-year survival is still not satisfied.^[17,18]^ Thus, it is very essential to diagnose LC at early stage, which can help to improve survival rate in such patients.

Published studies found that microRNA 25 (mRNA 25) expression in serum is associated with LC, which may help to diagnose patients at early stage.^[19,20,21,22,23,24]^ However, no systematic review and meta-analysis is conducted to assess the association between mRNA 25 expression in serum and LC. Thus, this study will systematically evaluate the association between mRNA 25 expression in serum and LC.

## 2 Methods and design

### 2.1 Study registration

This study protocol has been registered prospectively on INPLASY202040056. It was reported based on the preferred reporting items for systematic reviews and meta-analyses protocol 2015 statement.^[25]^

### 2.2 Objective

This study will investigate the association between mRNA 25 expression in serum and LC.

### 2.3 Inclusion criteria for study selection

#### 2.3.1 Type of studies

All case-controlled studies on exploring the association between mRNA 25 expression in serum and LC will be included. All studies will be considered without any language and publication status limitations.

#### 2.3.2 Type of participants

This study will include patients with histopathology-proven LC or normal participants for inclusion without restrictions of race, age, gender, and educational background.

#### 2.3.3 Types of index test

Experimental group: mRNA 25 expression in serum was detected in all patients with histopathology-proven LC.

Control group: mRNA 25 expression in serum was checked in the normal participants without LC.

#### 2.3.4 Type of outcome measurements

The primary outcome is mRNA-25 expression (as measured by real-time polymerase chain reaction test).

The secondary outcomes are relevant LC parameters, including pathological types, tumor-node-metastasis stages, lymph node metastasis, and tumor markers.

### 2.4 Data sources and search strategy

We will retrieve Cochrane Library, PUBMED, EMBASE, Web of Science, Allied and Complementary Medicine Database, VIP database, and China National Knowledge Infrastructure from inception to the present without limitations of language and publication status. The search strategy for PUBMED is created (Table 1). We will adapt similar search strategies and will apply them to the other electronic databases. We will include all eligible case-controlled studies that report association between mRNA 25 expression in serum and LC.

**Table 1:**
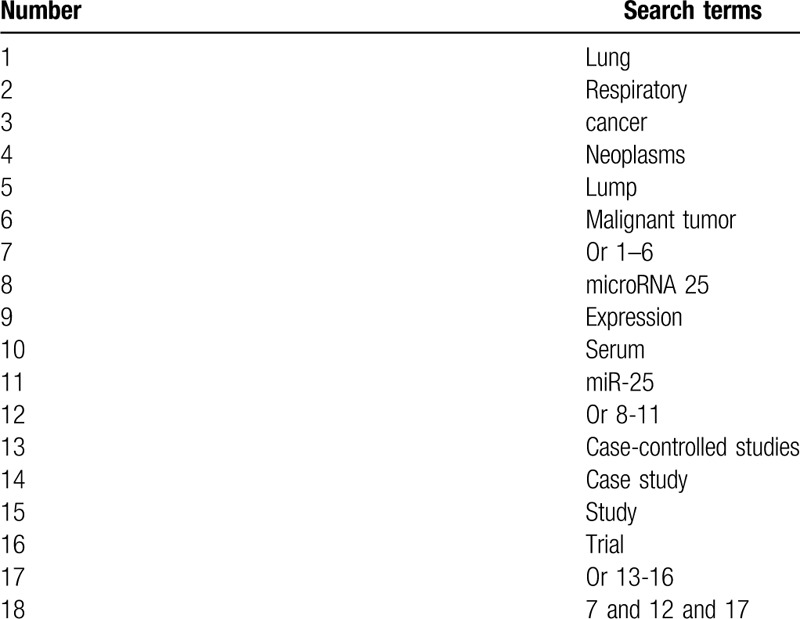
Search strategy for PUBMED.

In addition, we will also examine other literature sources, including conference proceedings and reference lists of related reviews.

### 2.5 Study selection and data collection

#### 2.5.1 Study selection

Two contributors will independently screen all titles/abstracts according to the predefined eligibility criteria. We will exclude all duplicated studies and irrelevant studies. After that, we will obtain and read the full manuscripts of the remaining studies against all inclusion criteria. In case of disagreements of study selection, a consensus will be reached with the help of another contributor. We will present selection process of study in a flowchart with detailed information.

#### 2.5.2 Data extraction

Two contributors will independently extract data according to the predefined and standardized data extraction sheet. Any conflicts between 2 contributors will be resolved by another contributor through consultation. The following data will be extracted from each trial: first author, journal, time of publication, region, race, age, sex, diagnostic criteria, inclusion and exclusion criteria, sample size, study design, types of targeted subjects, outcome indicators, and conflict of interest. If we identify any missing or insufficient information, we will contact original authors to obtain that information.

### 2.6 Quality assessment

Study quality of each included study will be evaluated using Newcastle–Ottawa scale.^[26]^ Two independent contributors will assess study quality, respectively. Any difficulties encountered between 2 contributors will be solved with another contributor.

### 2.7 Assessment of heterogeneity

Two contributors will identify heterogeneity across included studies using *I*^*2*^ statistic. *I*^*2*^ ≤ 50% exerts reasonable heterogeneity, and a fixed-effects model will be deployed. On the other hand, *I*^*2*^ > 50% means significant heterogeneity, and a random-effects model will be employed.

### 2.8 Statistical analysis

#### 2.8.1 Data synthesis

This study will utilize RevMan V.5.3 software for data synthesis and data analysis. We will estimate the treatment effect of continuous data as mean difference or standardized mean difference and 95% confidence intervals, and dichotomous data as risk ratio and 95% confidence intervals. We will carry out a meta-analysis if sufficient data are collected from eligible studies with homogeneity. On the other hand, we will perform a subgroup analysis to examine the sources of heterogeneity. In addition, we will also conduct a narrative summary.

#### 2.8.2 Subgroup analysis

We will carry out subgroup analysis to check sources for significant heterogeneity based on the different types of study characteristics, study methods, and outcome indicators.

#### 2.8.3 Sensitivity analysis

We will perform sensitivity analysis to explore the robustness and stability of study findings by removing low quality studies.

#### 2.8.4 Reporting bias

We will conduct reporting bias by funnel plots and associated regression tests if more than 10 studies are included.^[27]^

### 2.9 Quality of evidence

The quality of evidence for each outcome will be assessed using the grading of recommendations assessment, development, and evaluation approach.^[28]^

### 2.10 Ethics and dissemination

This study will not require ethic approval, because this study will not use individual patient data. This study will be disseminated through a relevant peer-reviewed journal or a conference.

## 3 Discussion

Although previous studies have reported the association between mRNA 25 expression in serum and LC,^[17,18,19,20,21,22,23,24]^ no systematic review and meta-analysis has been conducted to explore this topic. This study will be the first one to investigate the current existing evidence to identify the association between mRNA 25 expression in serum and LC. It is expected that the results of this systematic review will be an essential step towards informing the association between mRNA 25 expression in serum and LC, which may benefit both for clinical practice and further research.

## Author contributions

**Conceptualization:** Peng Luo, Shu-Min Li, Yu-Tong Xing, Jia-Bin Sun.

**Data curation:** Peng Luo, Feng Qiao, Peng-Hui Dou.

**Formal analysis:** Peng Luo, Feng Qiao, Yu-Tong Xing, Gang Zhou, Su-Kun Xu, Jia-Bin Sun.

**Investigation:** Jia-Bin Sun.

**Methodology:** Peng Luo, Feng Qiao, Peng-Hui Dou, Shu-Min Li, Tian-Lu Zhang, Gang Zhou, Su-Kun Xu.

**Project administration:** Jia-Bin Sun.

**Resources:** Feng Qiao, Peng-Hui Dou, Shu-Min Li, Tian-Lu Zhang, Yu-Tong Xing, Gang Zhou, Su-Kun Xu.

**Software:** Peng Luo, Feng Qiao, Peng-Hui Dou, Shu-Min Li, Tian-Lu Zhang, Yu-Tong Xing, Gang Zhou, Su-Kun Xu.

**Supervision:** Jia-Bin Sun.

**Validation:** Peng Luo, Feng Qiao, Shu-Min Li, Tian-Lu Zhang, Gang Zhou, Jia-Bin Sun.

**Visualization:** Peng Luo, Feng Qiao, Peng-Hui Dou, Yu-Tong Xing, Gang Zhou, Jia-Bin Sun.

**Writing – original draft:** Peng Luo, Peng-Hui Dou, Shu-Min Li, Tian-Lu Zhang, Yu-Tong Xing, Su-Kun Xu, Jia-Bin Sun.

**Writing – review and editing:** Peng Luo, Feng Qiao, Shu-Min Li, Gang Zhou, Jia-Bin Sun.
